# Aerosol Filtration Performance of Solution Blown PA6 Webs with Bimodal Fiber Distribution

**DOI:** 10.1021/acsomega.2c05449

**Published:** 2022-12-08

**Authors:** Melike Gungor, Sule Selcuk, Ali Toptas, Ali Kilic

**Affiliations:** † TEMAG Lab., Textile Technol. and Design Faculty, Istanbul Technical University, Istanbul34437, Turkey; ‡ Safranbolu Vocational School, Karabuk University, Karabuk78050, Turkey; § Areka Advanced Technologies Ltd. Co., Istanbul34467, Turkey

## Abstract

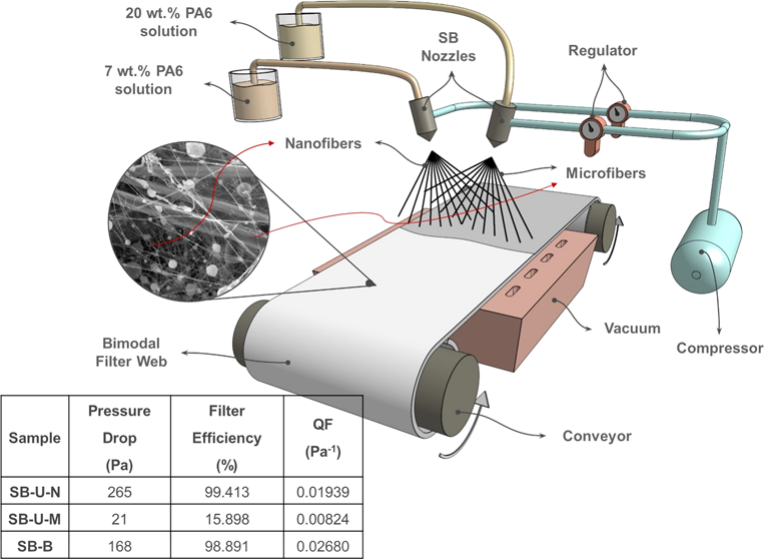

A bimodal web, where both nanofibers and microfibers are present and distributed randomly across the same web, can deliver high filter efficiency and low pressure drop at the same time since in such a web, filter efficiency is high thanks to small pores created by the presence of nanofibers and the interfiber space created by the presence of microfibers, which is large enough for air to flow through with little resistance. In this work, a bimodal polyamide 6 (PA6) filter web was fabricated via a modified solution blowing (m-SB) technique that produced nanofibers and microfibers simultaneously. Scanning electron microscope (SEM) images of the webs were used to analyze the fiber morphology. Additionally, air permeability, solidity, porosity, filtration performance, and tensile strength of the samples were measured. The bimodal filter web consisted of nanofibers and microfibers with average diameters of 81.5 ± 127 nm and 1.6 ± 0.458 μm, respectively. Its filter efficiency, pressure drop at 95 L min^–1^, and tensile strength were 98.891%, 168 Pa, and 0.1 MPa, respectively. Its quality factor (QF) and tensile strength were 0.0268 Pa^–1^ and 0.1 MPa, respectively. When compared with commercially available filters, the bimodal web produced had superior filter performance, constituting a suitable alternative for air filter applications.

## 1 Introduction

Supplying clean air to people, buildings, machines, etc. is a vital task due to increasing pollution, and it has become even more critical due to more frequent pandemics in recent years. Nano- or microscale particles that cause air pollution, named particulate matter, are classified as PM10, PM2.5, PM1, and PM0.3. There are two major types of filter media, namely, membrane filters and fibrous filters.^1^ The latter are preferred more due to the relatively low pressure drop, high filter efficiency, light structure, high porosity, high gas penetration, high surface-to-volume ratio, and low cost.^2^

An ideal air filter medium delivers high performance, where the filter efficiency is high and the pressure drop is low. In general, a filter medium with small pores has high filter efficiency, but the small pores block airflow through the filter as well, resulting in a high pressure drop. Generally, an air filter medium with large pores, on the other hand, provides a low pressure drop, resulting in a very low filter efficiency. However, conventional spun-melt fibrous filter media do not exhibit high submicron particle capture efficiency since they have large pores due to their fibers being on the order of micrometers.^3−5^ They are predominantly produced by the spun-bonding and melt blowing (MB) technique, whose throughput rates are relatively high, making them available at low cost.^6^ Filter efficiency of microfibers can be increased by increasing the thickness, which in turn results in poor air permeability,^7^ or by charging the web by methods such as corona discharge,^8,9^ triboelectrification,^10^ and hydrocharging and thermal polarization.^11^ Such charged filters, known as electret filters, capture the particles to be removed in the air by electrostatic forces.^12,13^ The major drawback of electret filters, however, is that they lose their charge in time,^13^ which dramatically reduces the filter efficiency. In the case of nanofibrous filter media, the filter efficiency can be very high due to the high surface-area-to-volume ratio^14,15^ and most of them do not rely on electrification. However, filters with fibers thinner than 60–65 nm tend to have high solidity, which slows down the airflow substantially, resulting in high pressure drop and premature clogging.^16^ As far as strength of the webs is concerned, microfibrous webs perform considerably better than nanofibrous ones due to their rigidity. Therefore, bimodal webs have been developed to produce an ideal air filter by combining nanofibers and microfibers together in one filter medium. Strong and efficient filter media with low pressure drop can be obtained thanks to bimodal fiber distribution.^17,18^ They have a wide range of Knudsen numbers because of the presence of both micro- and nanofibers.^19^ Consequently, air flows through such webs with less resistance.

Early research on bimodal filters were mainly simulation- or theoretical-orientated.^20,21^ In their simulation study, Fotovati et al. reported that smaller particles were captured less as the ratio of the thick fiber in bimodal filter webs increased.^21^ Several techniques have been used to fabricate bimodal webs, and MB has been one of them. In one MB arrangement, two polymers with different melting points and molecular weights were spun from two separate extruders.^22^ In another study using the MB method, polypropylene (PP) and polystyrene (PS) polymers with different chemical structures were fed together to one extruder to obtain variable polymer melt viscosity.^18^ Additionally, island-in-the-sea type of MB production was also used to produce bimodal webs.^23^ In the literature, there are bimodal works where solutions with different concentrations^24^ or different types of polymers^25−27^ were spun by evolution strategy (ES), producing structures with nanofibers and microfibers in different layers. In the study of Deng et al., polylactic acid (PLA) solutions were prepared at 8, 10, 12, and 14 wt % concentrations, which led to nanofibers having average diameters of 300, 740, 770, and 1300 nm. The feed rate and electrical voltage were 3.2 mL h^–1^ and 15 kV, respectively. Since the most homogeneous production was obtained from the 12 wt % PLA solution, the nanofiber production was carried out on recycled polyester (R-PET) microfibrous nonwoven fabric. The fiber diameter distribution of the fabric of this production was between 1.2 and 3.4 μm, and the pores were between 1.3 and 5 μm. The bimodal fabric obtained had a filter efficiency of 99.992% at a face velocity of 32 L min^–1^, a pressure difference value of 201.11 Pa, and a quality factor (QF) value of 0.047 Pa^–1^.^27^ The high filter efficiency obtained in the study of Deng et al. was partially due to the electrical charges imparted during electrospinning. In a one-step ES bimodal study conducted in 2015, PA6-based nanofiber/-net membranes were produced to obtain a suitable combination of two-dimensional (2D) ultrathin nanonets (∼20 nm) and stable cavity structures for improved filtration performance. In that work, the polymer was dissolved in formic acid and acetic acid, whose different weight ratios were tried. Depending on the polymer concentration, the diameter of the scaffold nanofibers varied between 134 and 890 nm and the coverage rates of nanonets varied between 8 and 98%. Filter efficiency and pressure drop were found to be 99.995% and 111 Pa at a face velocity of 32 L min^–1^, respectively.^28^ The electrospinning method was also used in that study, and, for that reason, the high filter efficiency obtained can be attributed to the electrical charges on the fabric. The same group carried out a similar study with the poly(*m*-phenylene isophthalamide) (PMIA) polymer in 2017.^29^ Subsequently, thick and thin fibers, made of two different polymers PLA/PMMA(poly(methyl methacrylate)), were produced.^30^ In that study, the ratio of fine to thick fiber decreased with increasing concentration. The ideal results were obtained when the concentration was 15 wt % and the corresponding fiber diameters were found to be 550 and 1300 nm for the thin and thick fibers, respectively, giving a thickness ratio of 2.4 approximately.^30^ In that bimodal study, the size difference between the nano- and microfibers obtained was not substantial. In a similar study to ours, polyacrylonitrile (PAN)-based bimodal and unimodal mats with the same weight-averaged diameter and similar packing density were spun by ES and tested and the bimodal ones were found to exhibit higher quality factors.^24^ Therefore, the modified solution blowing (m-SB) technique constitutes an innovative approach. In an earlier work of ours, we added glass particles to a polyamide 6 (PA6) solution to increase interfiber distances in the nanofibrous web and, hence, decrease the filter pressure values.^31^ In our current work, however, we took the approach of increasing the interfiber distances by means of inserting a small amount of microfibers, approximately 10% in weight, among nanofibers, creating bimodal webs. Polyamide 6 (PA6), which is a widely used polymer in filter and membrane technologies, has been selected for this study mainly due to its toughness, flexibility, and easy processing.^32^ In addition, it is wear-resistant and exhibits high tensile and impact strength. PA6 is also a nontoxic material that can be mixed with carbon or glass fibers for improved performance.^33^ Additionally, it is biodegradable and biocompatible and has superior mechanical properties due to intermolecular hydrogen bonds.^34^ Unlike polyethylene oxide and poly (vinyl alcohol), PA is water resistant.^35^ Since PA is soluble in formic or formic/acetic acid, this makes it a more environmentally friendly option, based on EU directive 67/548/EEC, than dimethylformamide (DMF), which is toxic.^36^

**Figure 1:**
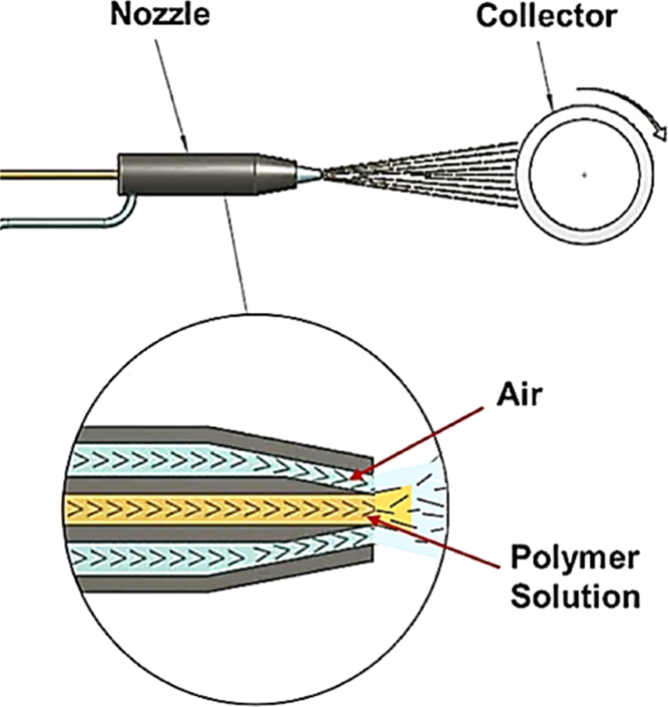
Schematic drawing of the solution blowing (SB) apparatus.

For our current study, we developed an m-SB setup, illustrated in Figure 8-1. In the m-SB, the polymer solution fed through a needle is subjected to a coaxial air jet, which causes evaporation of the solvent of the polymer solution rapidly.^37^ The remaining polymer jet is stretched because of the air drag forces and then finally lands on a rotating vacuum collector.^38^ The throughput rate of the regular SB technique is 30 times higher than that of the regular ES technique.^39^ It is possible to produce fibers finer than 100 nm by SB without deploying a high voltage. Absence of electricity makes it possible to access the nozzle(s) during production in the case of any problems. Furthermore, neither the polymer nor the solvent has to be conductive in SB, unlike ES. The morphology of the solution blown mats is mostly affected by material parameters such as polymer concentration, solution viscosity, molecular weight and molecular weight distribution of the polymer, solvent evaporation rate, and solution feed rate.^38^ Air pressure is the dominant process parameter, while humidity also has considerable effects on the web structure. Another important process parameter in SB is the nozzle-to-collector distance, which heavily determines bead formation and homogeneity of the structure Figure 1.^38^

In this work, PA6-based bimodal webs with micro- and nanofibers were manufactured by the m-SB technique to obtain a mechanically strong fiber medium that can deliver high filtration efficiency and low pressure drop at the same time. Additionally, two unimodal samples, one nanofibrous and the other microfibrous, were manufactured to compare with the bimodal sample. Various polymer concentrations, feed rates, and air pressure values were tried to obtain optimum fibrous webs. SEM analysis was deployed to characterize the webs qualitatively and also used to determine the fiber diameter. In addition, air permeability, solidity, porosity, pressure drop, filter efficiency, tensile strength, and modulus of elasticity of the webs produced were measured. Finally, the bimodal filter sample developed was compared with five commercial filters.

## 2 Materials and Methods

### 2.1 Materials

Polyamide 6 (PA6, BASF Ultramid B27 02, 1.14 g cm^–3^, 34 MFI) was used as the polymer precursor. Acetic acid (AA, 99.9% purity, TEKKIM) and formic acid (FA, 99.9% purity, Merck) were the cosolvents for PA6 with a ratio of 2:1, respectively. The fibers produced were collected onto 13 gsm polyester (PET) spun-bond support fabrics (Mogul Company).

### 2.2 Fiber Web Production

Fibrous filter samples were produced by a pilot line SB device (Aerospinner P1.0, AREKA Advanced Technologies Ltd. Co., Turkey). For producing unimodal webs, a basic setup of the SB device, with a single nozzle and a syringe pump, was used. For producing bimodal webs, a modified setup of the SB device was used, where two polymer solutions with different polymer concentrations were simultaneously fed through two separate nozzles by two separate syringe pumps and the compressed air was introduced from one regulator. The two nozzles were positioned in such a way that they generated two jets converging on the collector. A 22-gauge needle nozzle was selected since SB needles with larger diameters prevent compressed air from overcoming the surface tension of the polymer concentration^38^ and smaller diameters result in low yields. The nozzle-to-collector distance was 40 cm since it was found earlier to be at least 30 cm to allow solvent evaporation.^38^ Also, single nozzle devices often produce a higher basis weight at the center of the solution jet from the nozzle and a lower basis weight toward the edge of the solution jet, which results in nonuniform web properties. Thus, a distance of 40 cm is the ideal distance to obtain a more uniform web. The fibers were collected by a vacuum collector onto which a spun-bond nonwoven was wound. The collector vacuum rate was 50 L min^–1^. Solution viscosity was measured prior to fiber production in every trial.

The fiber production trials comprised preliminary experiments (unimodal only) and the main experiments (bimodal and unimodal). The preliminary experiments were conducted to determine the values of process parameters to be used in the subsequent main experiments, which involved producing unimodal and bimodal webs and comparing them. Details of the main experimental work are summarized in Table 1.

**Table 1:** Production Parameters for the Main Experiments

sample	solution concentration nozzle 1 (%)	solution concentration nozzle 2 (%)	amount of 7 wt % solution used (mL)	amount of 20 wt % solution used (mL)
SB-U-N	7		5	
SB-U-M		20		5
SB-B	7	20	2.5	2.5

In the preliminary experiments, three levels of solution concentrations (7, 13, and 20 wt %), three levels of feeding rates (5, 10, and 15 mL h^–1^), and two levels of air pressure (1 and 2 bar) were tried, and unimodal webs were obtained from 1 mL of polymer solution. Table S1 includes the details of the preliminary experiments. After evaluating the preliminary experiments, 2 bar and 5 mL h^–1^ were found to be the suitable levels for air pressure and feed rate, respectively. In addition, concentrations of 7 and 20 wt % were found to be the suitable levels for producing unimodal webs with the finest fibers and unimodal webs with the coarsest fibers, respectively, in the main experiments. The idea in the main experiments was to produce the following three types of fibrous webs and compare them:

- Unimodal web 1: Nanofibrous web (SB-U-N)
- Unimodal web 2: Microfibrous web (SB-B-M)
- Bimodal fibrous web containing both nanofibers and microfibers in the same web (SB-B).

In the main experiments, 5 mL of polymer solutions was consumed in each unimodal production. Since a solution feed rate of 1:1 was found to give the optimum result in bimodal production,^24^ 2.5 mL of 7 wt % and 2.5 mL of 20 wt % solutions were used for the bimodal production.

## 3 Characterization

The fibrous samples produced were first characterized by qualitative SEM analysis and then their fiber diameters were measured. Subsequently, pressure drop, filter efficiency, solidity, air permeability, tensile strength, and modulus of elasticity of the webs with a homogeneous morphology were found.

### 3.1 Viscosity

Viscosities of all of the prepared PA6 solutions were measured by a viscometer (Rotational Viscometer, Fungilab, α Series) prior to fiber production and presented in Table S2.

### 3.2 Morphology and Fiber Diameter of the Preliminary and Main Samples

SEM images of the web samples, taken by Tescan Vega 3, were analyzed to characterize the webs qualitatively. In addition, ImageJ program was deployed to determine the fiber average diameter of the preliminary and main samples. Images were taken at 500×, 5000×, and 10,000× magnifications. Prior to scanning by the SEM, the samples were sputter-coated with gold-palladium (AuPd) using a fine coater.

Color map analysis was done manually using SEM images of the preliminary experiments. Also, average fiber diameters were obtained by measuring at least 100 fibers from the SEM images for both preliminary and main experiments. Fiber ratio in the bimodal samples was defined as the ratio of the number of fibers larger than 1 μm in diameter to that of the submicron fibers in the web.

### 3.3 Air Permeability Characteristics of Webs

The air permeability, which is related to thickness and porous structure of the web, is an important property of the filter medium in determining its filter performance^40−43^ as it gives an idea about the amount of air that passes through the filter fabric.^44^

The Prowhite Airtest II model air permeability device was used to measure the air permeability of the filter media produced according to ASTM D737, in which the the sample diameter is 38 cm^2^, the air pressure is 125 Pa, and the ambient temperature is 22 ± 2 °C.

### 3.4 Packing Density (Solidity) of Webs

In general, fabrics/webs/mats with high solidity deliver high airflow resistance and high filter efficiency.^45^ Solidity measurements were carried out to examine the filter performance of the webs in more detail. Solidity was calculated according to eq 1.^46,47^ The basis weight, fabric thickness of the webs, and density of the polymer used to produce the fibers are the governing factors in calculating solidity.
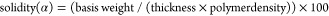
1

The overall porosity (ε) of the webs was calculated by eq 2.
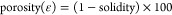
2

Basis weight measurements were done from 5 × 5 cm^2^ mats, which are peeled off from the spun-bond support fabric and weighed. Web thickness was measured by a digital comparator.

### 3.5 Pore Size of Webs

In fibrous filter webs, since the fiber diameter is a factor that determines the porosity of the webs, the porosity and pore size distribution of webs have a significant effect on pressure drop.^48^ The porometer instrument (POROLUX 100NW, Boynton Beach, FL) has a test capacity of pore size within 0.427–500 μm. Porofil with 16 dyn cm^–1^ surface tension was used as a wetting liquid for the analysis. First, the sample is impregnated with an inert, nontoxic wetting liquid. “Wet run” produces a “wet curve,” which represents the measured gas flow through the sample against the applied pressure. Then, the same method is used for a dry sample measurement. The “half-dry curve” is obtained by dividing the flow values of the dry curve by 2. In summary, from the wet curve, dry curve, and the half-dry curve data, information about the porous network are obtained.^49^

### 3.6 Filter Performance

Filter performance of the samples was determined using the filter test device (8130A model, TSI Inc.). Solid sodium chloride (NaCl) particles were generated from a NaCl solution with 2% mass concentration. Filter samples with 100 cm^2^ effective area were challenged against aerosols in the 0.26 ± 0.07 μm range at face velocity 15.83 cm s^–1^. The device measures pressure drop and filtration efficiency of the filter samples, which are the two main parameters that determine the quality of an air filter.^46^ The QF, which is a quantitative criterion to compare various nanofibrous filters, is calculated using eq 3
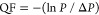
3where *P* is the penetration and Δ*P* is the pressure drop across the media.^50^ QF is considered the benefit-to-cost ratio of a filter, where “benefit” refers to filtration efficiency and “cost” refers to pressure drop.^51^

Another important parameter in analyzing filter performance is the Knudsen number (*Kn*) as it describes the airflow around the filtering fiber. This dimensionless number is defined in eq 4
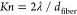
4where λ, which is equal to 65 nm at 298 K and 1 atm, is the molecular mean free path in air and *d*_fiber_ is the fiber diameter.^52^

### 3.7 Mechanical Performance of Webs

An Instron 4411 Universal Tensile Tester with a 50 N load cell was used to perform the tensile test of the webs. The webs to be tested were first peeled off from the nonwoven support layer. The samples were then cut into strips of 50 × 10 mm^2^, and then, the web thickness was measured at three different points of each sample to take the average. The gauge distance was 30 mm, and the test speed was 30 mm min^–1^. The samples were conditioned at 24 °C for 24 h prior to the tensile test.

## 4 Results and Discussions

### 4.1 Morphology of the Preliminary and Main Samples

**Figure 2:**
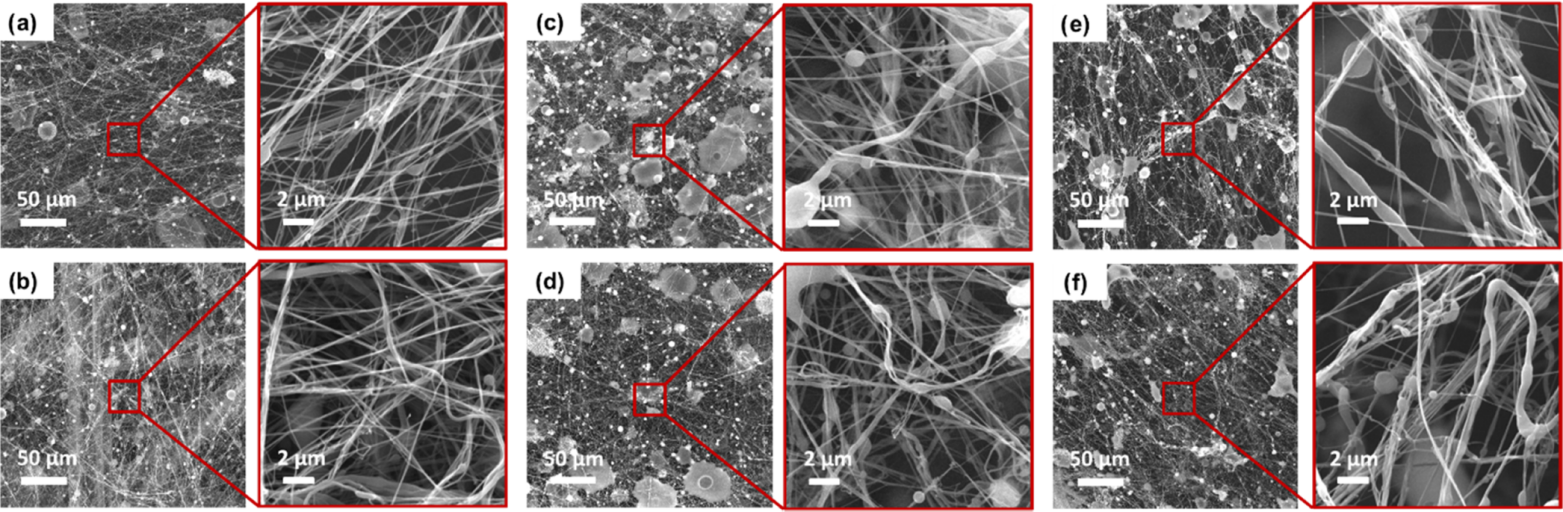
SEM images of the preliminary samples produced with 7 wt % solution: (a) SB 1.1, (b) SB 1.2, (c) SB 1.3, (d) SB 1.4, (e) SB 1.5, and (f) SB 1.6 (scale bars are 50 μm for big pictures and 2 μm for small pictures).

SEM images of the fibrous webs produced in the preliminary experiments are presented in Figure 2. The images reveal that samples with a low solution concentration (7 and 13 wt %), i.e., samples of between SB 1.1 and SB 1.12, exhibited beads of some sort. As seen in Table S2, solutions with a low concentration also have low viscosity. Bead formation in the case of inconsistency of the polymer solution jet was attributed to low viscosity.^53^ It was reported that more stable polymer solution jets were obtained with increasing viscosity.^54^ The samples of 20 wt % concentration solution, i.e., samples of between SB 1.13 and SB 1.18, seemed to have a rather thick fiber formation, which can be attributed to high surface tension. On the other hand, the air pressure used cannot stretch the polymer jet further due to the increased viscosity and this causes the formation of thick fibers.^28^ The webs have less dense structures with rather straight and beadless fibers, and their fiber diameters were on the micron scale, as expected. In some cases of the 20 wt % solution concentration, the polymer solution that cannot form fibers landed on the collector, creating a kind of droplet. In the case of high concentration and low air pressure, insufficient amounts of fibers were produced as the air pressure was not high enough to overcome surface tension. As in the cases of low air pressure, the increase in the feed rate in high-viscosity solutions also affects the fiber formation negatively due to the insufficient air pressure.

**Figure 3:**
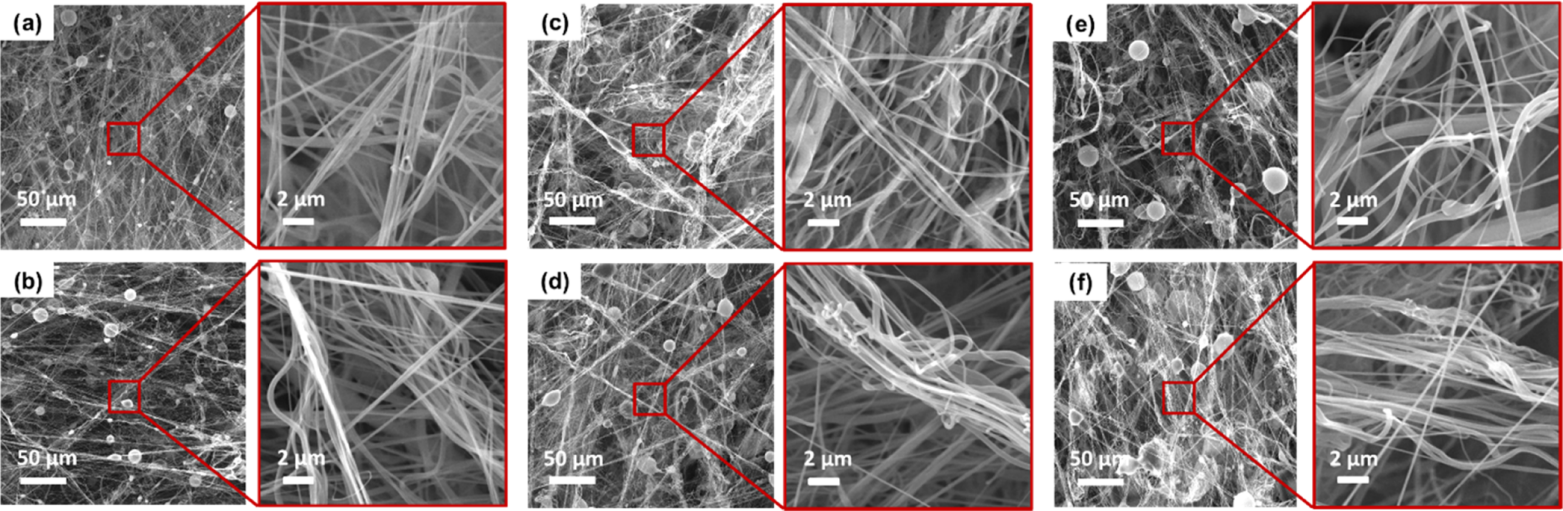
SEM images of the preliminary samples produced with 13 wt % solution: (a) SB 1.7, (b) SB 1.8, (c) SB 1.9, (d) SB 1.10, (e) SB 1.11, and (f) SB 1.12 (scale bars are 50 μm for big pictures and 2 μm for small pictures).

**Figure 4:**
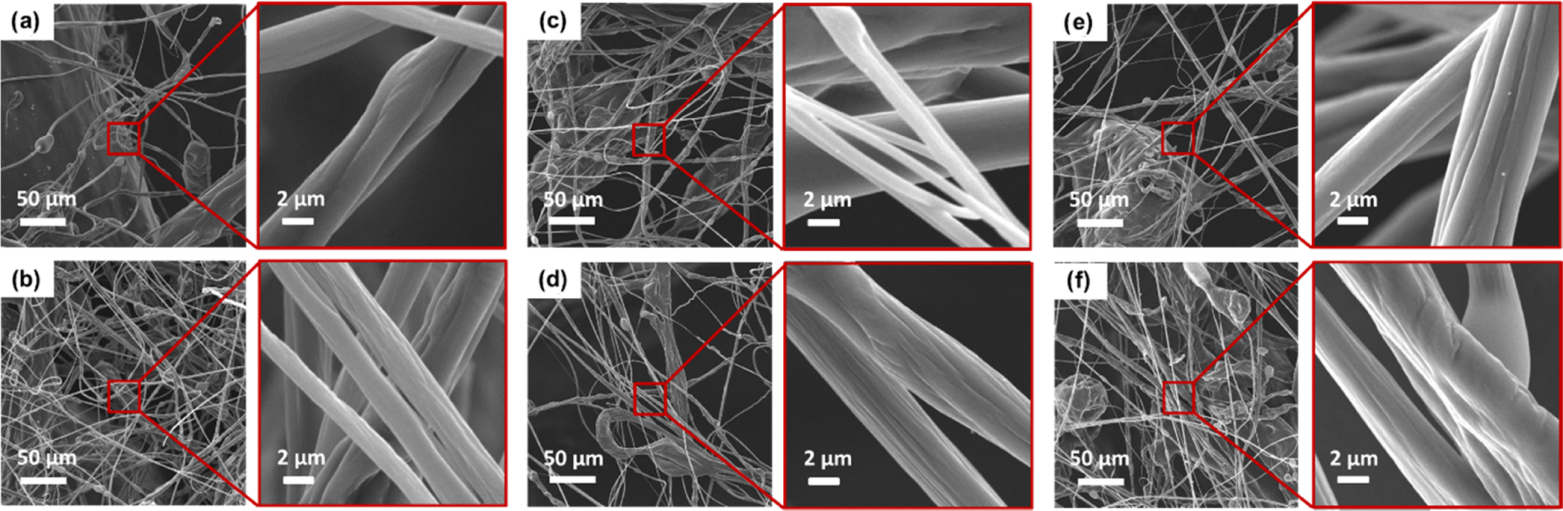
SEM images of the preliminary samples produced with 20 wt % solution: (a) SB 1.13, (b) SB 1.14, (c) SB 1.15, (d) SB 1.16, (e) SB 1.17, and (f) SB 1.18 (scale bars are 50 μm for big pictures and 2 μm for small pictures).

As far as the feed rate is concerned, all of the 5 mL h^–1^ runs exhibited structures with the fewest beads and droplets (Figures 2–4). It is evident in Figures 2 and 4 that entangled fibers, irregularities, and especially defects increased as the feed rate increased. With a concentration of 20 wt %, thicker and fewer fibers were obtained, especially in production with 1 bar air pressure (Figure 4).

**Figure 5:**
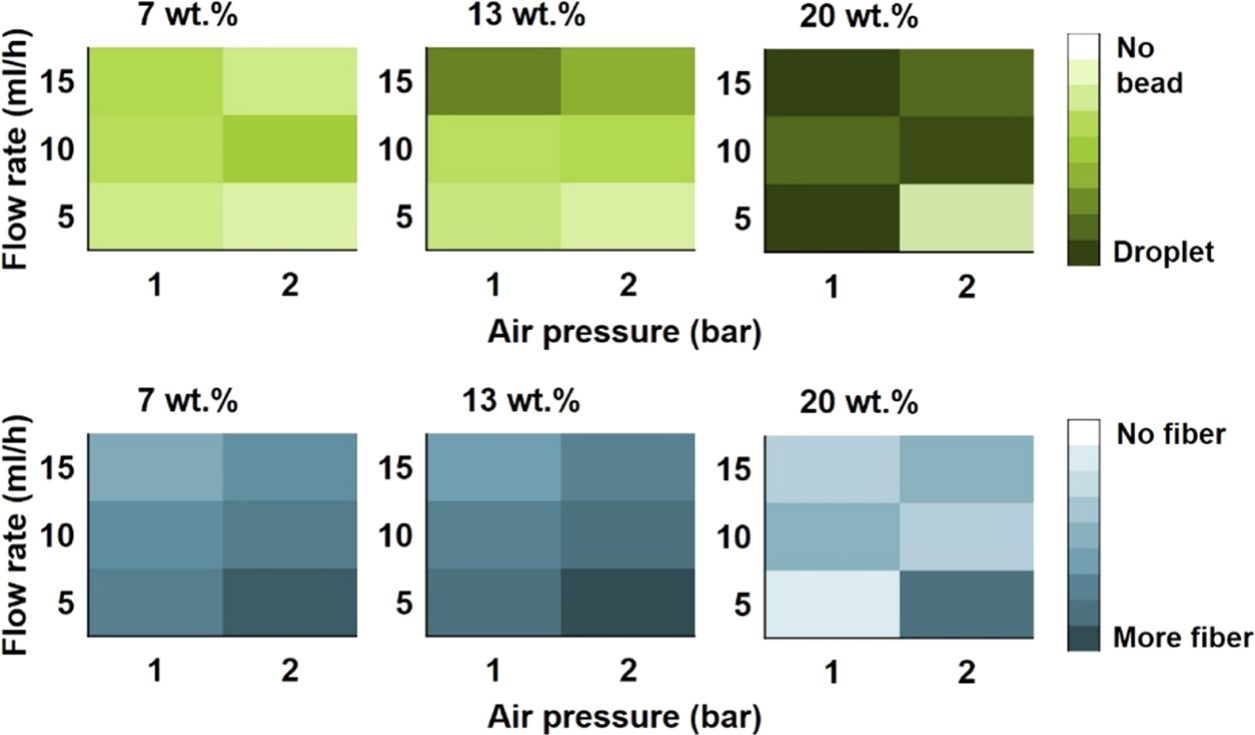
Colormaps of the preliminary experiments with reference to bead and fiber amount.

Figure 5 presents the color map analysis of the fibrous webs obtained in preliminary experiments. This analysis was done manually. It can be seen in the figure that as the concentration increased, darker shades of green became dominant, indicating structures with droplets. In the case of the 7 wt % concentration, there were fair amounts of beads but no droplets were present, leading to the conclusion that decreasing viscosity reduced the size of droplets into beads. It is also evident in the second row of the color map that the 7 and 13 wt % samples had almost the same amount of fiber. In the case of the 20 wt % sample, considerably fewer fibers were formed, which is evident from the lighter shades of blues. Decrease in the feeding rate and increase in air pressure seemed to reduce beads and droplets and, hence, increased the fiber formation.

**Figure 6:**
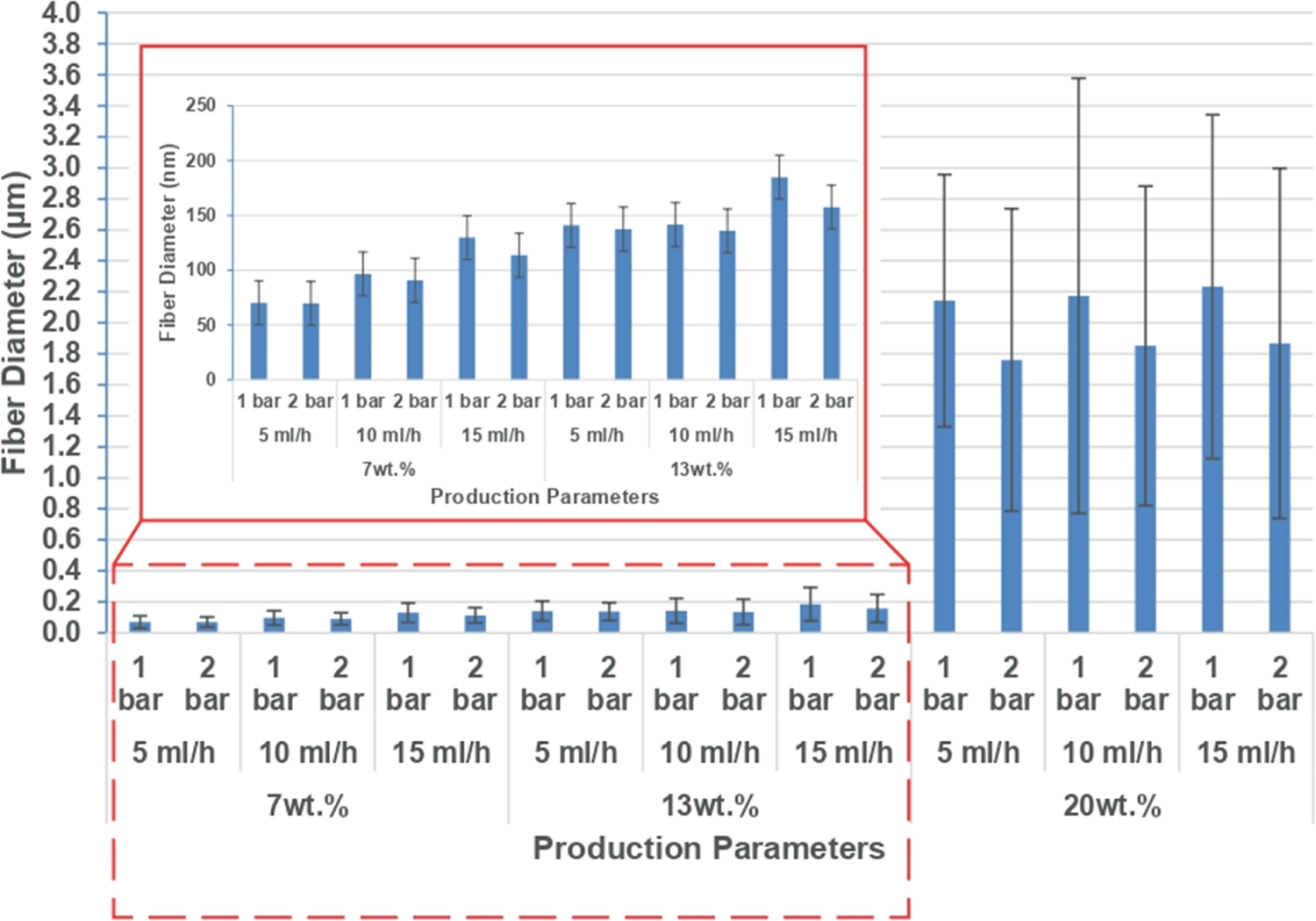
Fiber diameter vs production parameters in the preliminary experiments.

Effects of the three process parameters on fiber diameter are presented in Figure 6. It can be clearly seen that as the solution concentration increased, fibers become coarser, as expected. While fiber diameters of solutions with 7–13 wt % concentrations were 70 and 180 nm, respectively, a more dramatic diameter increase from 180 nm to 2.2 μm was recorded when the solution concentration was raised from 13 to 20 wt %. This jump in fiber diameter, which is more than 10 times, can be attributed to the fact that while the 7 wt % PA6 solution had a viscosity value of 71.6 mPa·s, viscosity of the 20 wt % solution was 1488.6 mPa·s.

Similar to the effect of concentration, increasing the feed rate also resulted in coarser fibers in general although this effect was less pronounced in the highest concentration runs (20 wt %). As far as air pressure is concerned, finer fibers seemed to be obtained at higher pressures and no significant changes in SEM images were observed. In the preliminary experiments, when the concentration was the highest (20 wt %) and the feed rate was the lowest (5 mL h^–1^), a higher air pressure, 2 bar as opposed to 1 bar, rendered efficient fiber formation.

**Figure 7:**
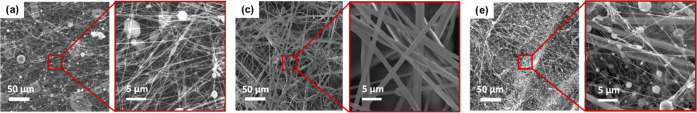
Scanning electron microscope images of bimodal PA6 fibers: (a) SB-U-N, (b) SB-U-M, and (c) SB-B.

Figure 7 shows SEM images of the webs produced in the main experiments. According to the images, SB-U-M consists of microfibers only and SB-U-N consists of nanofibers only but with beads due to low viscosity. Since the bead sizes were on the nanoscale, the web was still a nanofibrous structure. In the case of SB-B, both nanofibers and microfibers are present in the web. Since two separate nozzles were positioned in a way that they created two jets converging on the same point on the collector, the SB-B exhibited fine and coarse random distribution, creating a homogeneous structure with pores of various sizes. On the other hand, cross sections of the samples are given in Figure S1. According to these images, the thickness of the webs and the space between the fibers increase depending on the presence of microfibers in the webs.

**Figure 8:**
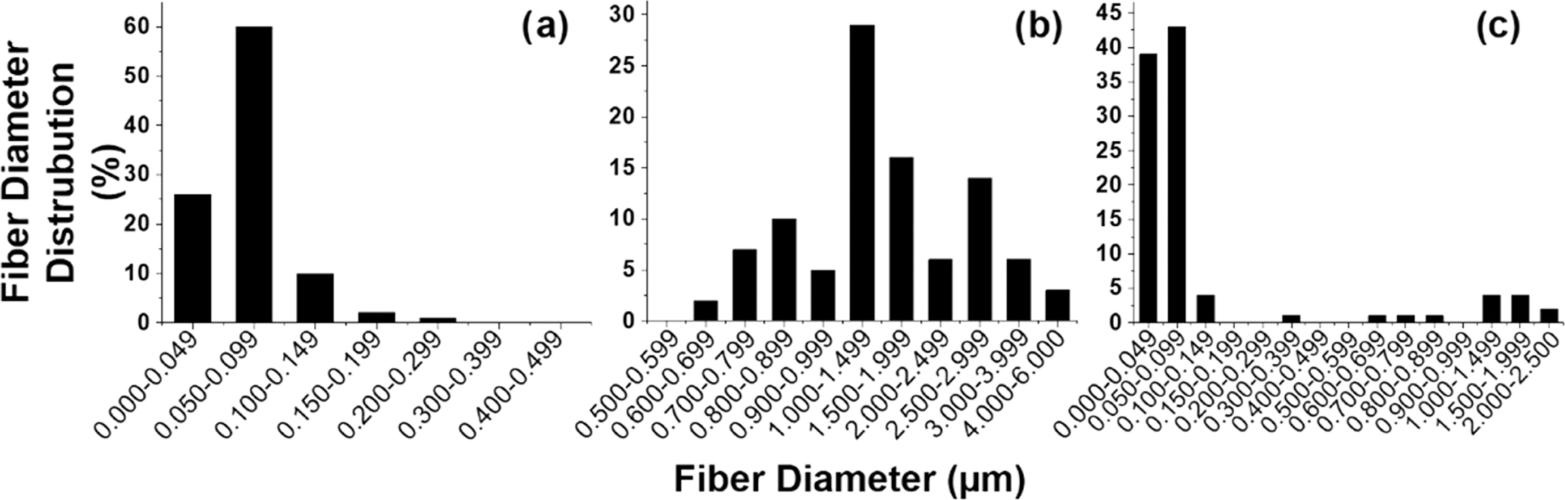
Fiber diameter distribution of (a) SB-U-N, (b) SB-U-M unimodal, and (c) SB-B bimodal webs.

Fiber diameter distributions of webs are presented separately in Figure 8. Average fiber diameters of SB-U-N and SB-B-M were measured to be 70 nm and 1.8 μm, respectively, while for SB-B, 10% of the fibers are on the microscale and 90% of the fibers are on the nanoscale.

### 4.2 Air Permeability of Webs

Air permeability and solidity results of the three samples are presented in Table 2, along with web thickness and weight measurements, which were used to calculate solidity using eq 1. As seen in the table, SB-U-N had the smallest air permeability of 23.79 m^3^ h^–1^, which could be attributed to its small pores formed by the very fine nanofibers. SB-U-M, on the other hand, had the highest air permeability of 214.08 m^3^ h^–1^, and this could be attributed to the larger interfiber space created by the coarser microfibers in the web. In the case of SB-B, where microfibers and nanofibers were incorporated in the same web, air permeability was 39.08 m^3^ h^–1^, which was somewhere between those of SB-U-M and SB-U-N, as expected. Increase in air permeability was rather limited since only 10% of the fibers in SB-B were on the microscale. Solidity of the bimodal web is distinctly smaller than the unimodal webs, suggesting that the strategy to loosen the otherwise tightly packed structures of all-nanofiber webs by means of inserting occasional microfibers among nanofibers seems to serve the purpose.

**Table 2:** Solidity and Air Permeability Results of SB-U-N, SB-U-M Unimodal, and SB-B Bimodal Webs

samples	thickness (μm)	basis weight (gr m^–2^)	solidity (%)	porosity (%)	air permeability (m^3^ h^–1^)
SB-U-N	37.25 ± 13.21	3.427	8.07	91.93	23.786 ± 1.311
SB-U-M	181.40 ± 15.26	18.021	8.71	91.29	214.075 ± 0.441
SB-B	54.75 ± 12.15	3.216	5.15	94.85	39.077 ± 1.013

### 4.3 Packing Density of Webs

As seen in eq 1, packing density is governed by web thickness and basis weight. Theoretically, solidity of webs produced from the same polymer with the same concentration decreases with increasing thickness.^55^ However, this is not the case for our samples of SB-U-N and SB-U-M, which are made of polymer solutions with different concentrations. Since SB-U-M had a higher polymer concentration, it had more mass than SB-U-N, and this resulted in SB-U-M having a higher mass-to-volume ratio. SB-B exhibited a similar structure to SB-U-N as 90% of the SB-B consisted of nanofibers. The presence of microfibers in SB-B seemed to reduce the packing density (Table 2).

**Figure 9:**
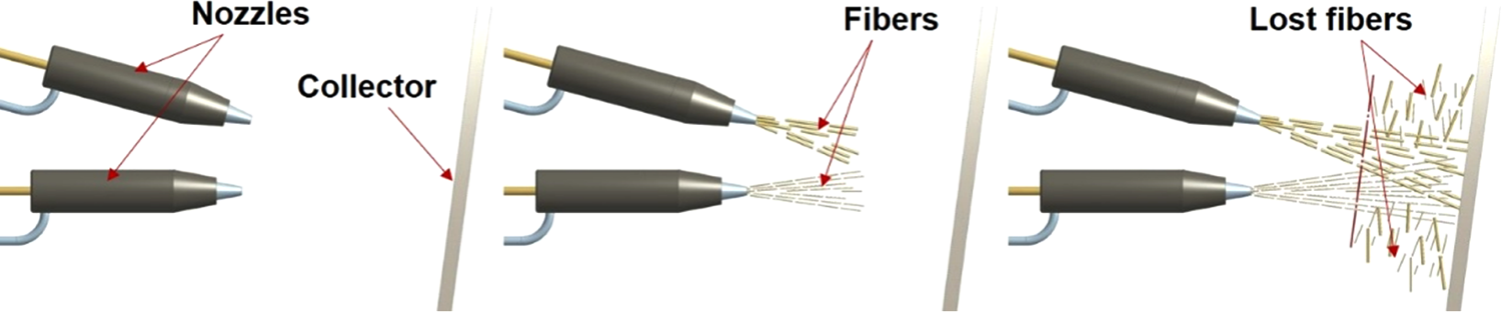
Schemas of producing bimodal webs and losing fibers in the m-SB method.

It should be added that in producing SB-B, two nozzles were positioned in such a way that at a given time, the two separate jets hit the same point on the collector, approximately doubling the pressure on that point, which may have caused some fiber loss in the web, hence resulting in a smaller basis weight than expected. This situation, which occurred during production, is illustrated in Figure 9.

### 4.4 Pore Size of Webs

Table 3 shows the fiber diameter, minimum, mean, and maximum pore size of the webs. The average fiber diameters and the mean pore sizes of the samples are also shown with the help of graphics in Figure S2. Also, flow/pressure graphs of the webs are given in Figure S3. In Figure S3, the wet curve starts with the bubble point (max. pore).^56^ According to Table 3, the SB-U-N sample has the smallest pore size with a size of 2.59 μm, while the SB-U-M sample has the largest (26.67 μm) pore size. Accordingly, the difference between the minimum and maximum pore size of the SB-B bimodal sample is the largest. The average diameter of fibers below 1 μm in the SB-B sample is about 10 nm thicker than that of the SB-U-N sample. It is thought that this situation causes the minimum pore size of the SB-B bimodal sample to be approximately 800 nm higher than that of the SB-U-N sample. Since the diameter of the microfibers in the SB-B bimodal sample is almost the same as that of the SB-U-M sample, the maximum pore size values are also almost the same. In general, the average pore sizes of the unimodal and bimodal samples are directly proportional to the average fiber diameters.

**Table 3:** Pore Sizes of the SB-U-N, SB-U-M Unimodal, and SB-B Bimodal Webs

samples	mean fiber diameter (μm)	smallest pore size (μm)	mean pore size (μm)	bubble point pore size (μm)
SB-U-N	0.07 ± 0.03	2.01	2.59	8.05
SB-U-M	1.76 ± 0.97	16.09	26.67	32.19
SB-B	0.23 ± 0.49	2.89	6.29	32.17
	0.08 ± 0.01	1.59 ± 0.46		

### 4.5 Filtration Performance of Webs

Knudsen numbers and filtration performance data of a single layer of the samples produced in the main experiments are presented in Table 4. It shows that SB-U-N achieves the highest filter efficiency of 99.413% due to its very fine fibers, which are 70 nm in diameter on average. However, its pressure drop was found to be as high as 265 Pa, which was expected from its impenetrable fibrous network, which is evident in its high solidity of 8.07%. In the case of SB-U-M, a very low pressure drop of 21 Pa but an extremely low filter efficiency of 15% as well were measured, which could be attributed to larger interfiber spaces formed by the larger fibers, which are 1.59 μm in diameter on average. As for SB-B, it delivered a considerably high filter efficiency of 98.89% at the expense of a pressure drop of 168 Pa. The bimodal sample, SB-B, delivered the best filtration performance, QF, of 0.02680, compared with the two unimodal samples.

**Table 4:** Knudsen Number and Filter Performance Data of the Bimodal and Unimodal Webs

sample	pressure drop (Pa)	filter efficiency (%)	Kn			QF (Pa^–1^)
SB-U-N	265	99.413	1.865			0.01939
SB-U-M	21	15.898	0.074			0.00824
SB-B	168	98.891	0.560	1.595	0.082	0.02680
			for microfibers	for nanofibers	for bimodal	

According to the airflow categorization based on the Knudsen number (Figure 2), flow regimes in SB-U-N and SB-U-M are transitional flow and slip flow, respectively. In the case of SB-B, however, this categorization is not clear-cut since this bimodal sample contains both microfibers with an average fiber diameter of 1.59 μm, which corresponds to slip flow, and nanofibers with an average fiber diameter of 81 nm, which corresponds to transitional flow.

**Figure 10:**
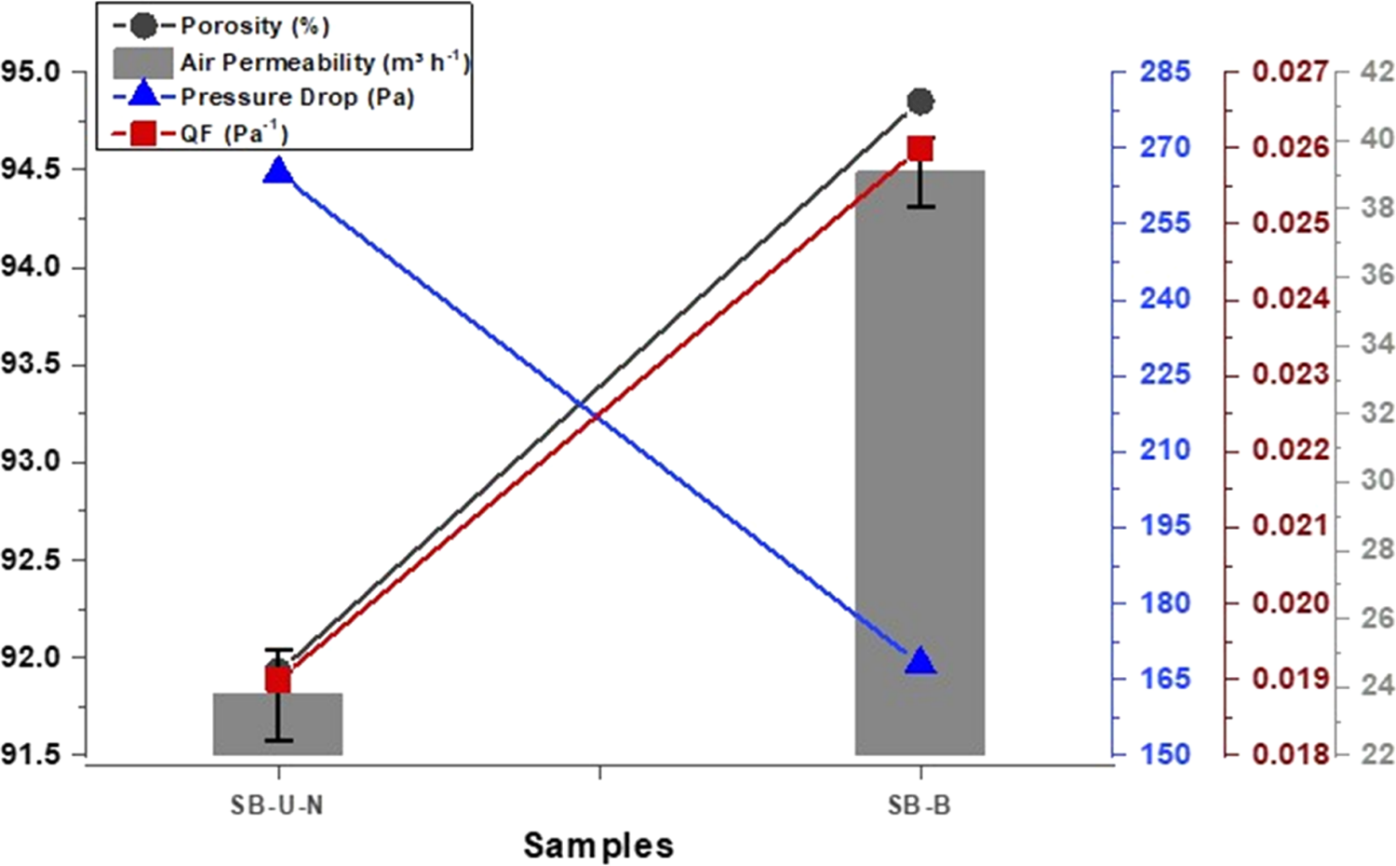
Comparative porosity, air permeability, and pressure drop graphs of the SB-U-N unimodal and SB-B bimodal webs.

Figure 10 contains porosity, air permeability, pressure drop, and QF data of SB-U-N and SB-B webs to compare them due to having approximately the same structure. It shows that the porosity of SB-B is higher than that of SB-U-N. The fact that SB-B achieves a lower pressure drop of 168 Pa and a higher quality factor of 0.026 Pa^–1^ suggests that the porous structure of the bimodal web is able to deliver a good overall filter performance without compromising the filter efficiency.

Finally, SB-B was tested along with five commercial filters (kindly provided by MGT Filters, Turkey) at a flow rate of 95 lt min^–1^, and the results are summarized in Table 5. In terms of quality factor, SB-B recorded a minimum 3 times better filtration performance than all of the commercial filters. When compared with the three F-filters, SB-B achieved the highest filter efficiency despite its extremely low basis weight. H13 recorded a very high filter efficiency of 99.93% but at the expense of a very high pressure drop of 874 Pa, and U15 also exhibited a similar performance to H13.

**Table 5:** Filtration Performance of the Bimodal Filter and Other Commercial Filters at a Flow Rate of 95 L min^–1^

filters	basis weight (g m^–2^)	pressure drop (Pa)	filter efficiency (%)	QF (Pa^–1^)
SB-B	3.2 ± 0.5	168	98.89	0.02680
F7	70.3 ± 0.9	76	38.63	0.00642
F8	77.9 ± 1.0	116	64.24	0.00840
F9	71.5 ± 1.2	172	75.26	0.00812
H13	80.3 ± 1.2	874	99.93	0.00831
U15	78.0 ± 0.9	1155	99.99	0.00797

### 4.6 Mechanical Performance of Webs

**Figure 11:**
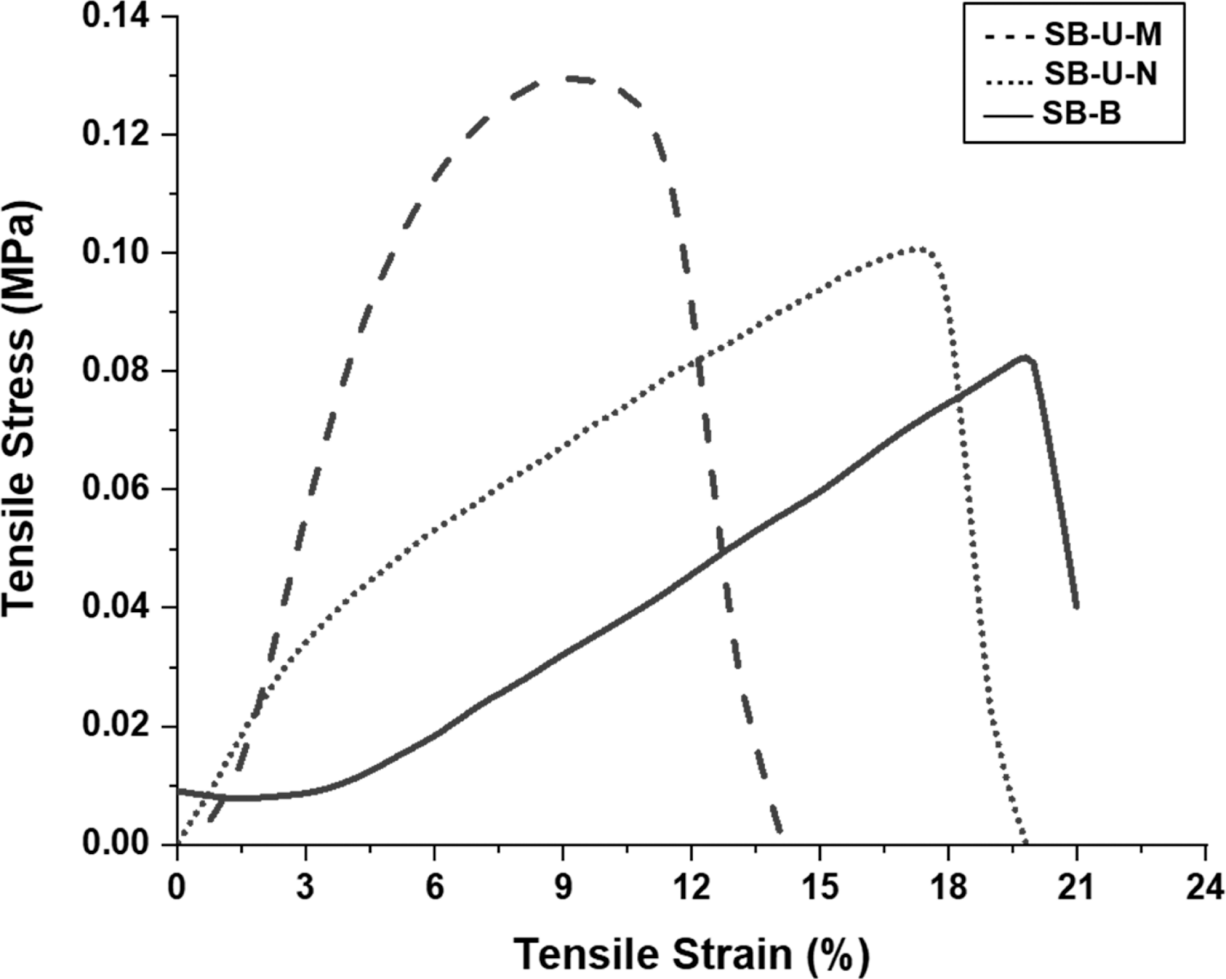
Stress–strain curves of SB-U-N, SB-U-M unimodal, and SB-B bimodal webs.

Figure 11 shows the tensile behavior of the three samples. All of the samples exhibited ultimate strength that is proportional to their fiber diameter. When compared with SB-U-N, the tensile strength of SB-B is 18% higher than that of SB-U-N, leading to the conclusion that filling microfibers into a nanofibrous web can reinforce the web structure. This 18% improvement is particularly valuable in practice since nanofibers are inherently difficult to handle due to their fine and delicate structure.

As far as the shape of the graphs is concerned, SB-B exhibited a stepwise rise as opposed to the almost continuous rise experienced by the unimodal samples. This intermittent rise in the bimodal web can be considered an indication of the dual nature of the web, where two groups of fibers, namely, coarse and fine fibers, are present and carry the tensile load in two different ways. The mechanical performance values are provided in Table S3 in detail.

## 5 Conclusions

Fibrous filters with ultrafine fibers exhibit superior performance for capturing particulate matter efficiently; however, such fine fibers usually flock closely, hence creating a dense structure with small pores, which causes high pressure drop. To circumvent this difficulty and still maintain a high capturing efficiency, the dense structure is needed to be loosened by creating larger pores as well within the nanofibrous web. For this purpose, the bimodal web approach was taken in this study, from which the following conclusions were drawn.

- PA6-based bimodal webs, which have both nano- and microfibers randomly distributed in the web, can be produced by the solution blowing technique. Of the total fibers, 90% are of nanofibers with an average fiber diameter of 81.5 ± 127 nm. The remaining 10% of the fibers are of microfibers with an average fiber diameter of 1.6 ± 0.458 μm. The dual characteristics of the bimodal web are evident in the intermittent shape of the tensile test curve. The results are consistent with the theoretical and experimental research in the relevant literature. The tremendous effect of the simultaneous spinning of nano- and microfibers led to larger pores without sacrificing the particle capture performance.
- The porosity test supports the hypothesis by showing that the bimodal web has both small and large pores thanks to the nanofibers and microfibers existing in the structure.
- Solution blowing was shown to be a versatile system to produce webs of bimodal fiber distribution. The modification involves employing independently driven two nozzles through which polymers with different concentrations are fed simultaneously. The nozzles are arranged to generate two jets converging on the collector.
- Microfibers in the bimodal filter reduce pressure drop and improve strength, while nanofibers provided high filter efficiency. The bimodal filter medium has a lower pressure difference, which leads to lower energy consumption compared to commercial filters. A larger dust-holding capacity and longer filtration life can be expected as well.
- The bimodal web produced has a higher quality factor than both the unimodal nanofibrous web and the microfibrous web, leading to the conclusion that bimodal webs can combine the benefits of coarse and fine fibers.

## Supporting Information Available

Cross-section view of the main samples SB-U-N, SB-U-M, and SB-B; comparative fiber diameter and pore size graphs of SB-U-N, SB-U-M unimodal, and SB-B bimodal webs; flow/pressure graphs of SB-U-N, SB-U-M unimodal, and SB-B bimodal webs; preliminary sample codes and variable system parameters during production; viscosities of different concentrations of PA6 solutions; and mechanical performance values of the webs (PDF)
