# Supplementary material for: Aerosol Filtration Performance of Solution Blown PA6 Webs with Bimodal Fiber Distribution
Source: ACS Omega. 2022 Dec 8;7(50):46602–12. doi: 10.1021/acsomega.2c05449 (PMC9773963; doi:10.1021/acsomega.2c05449)
Supplement: Supplementary file 1 — ao2c05449_si_001.pdf [file ao2c05449_si_001.pdf]

Aerosol Filtration Performance of Solution Blown PA6 Webs with Bimodal Fiber Distribution

Melike GUNGOR [1, 3], Sule SELCUK[1], Ali TOPTAS[1, 2], Ali KILIC[1, 3, *]

[1]TEMAG Lab., Textile Technol. and Design Faculty, Istanbul Tech. Univ., Istanbul, Turkey

[2]Safranbolu Vocational School, Karabuk Univ., Karabuk, Turkey

[3]Areka Advanced Technologies Ltd. Co., Istanbul, Turkey.

*alikilic@itu.edu.tr

Supporting Information

Figure S1. Cross section view of main samples a) SB-U-N, b) SB-U-M, and c) SB-B (The scale of the SEM image is 100 μm.).

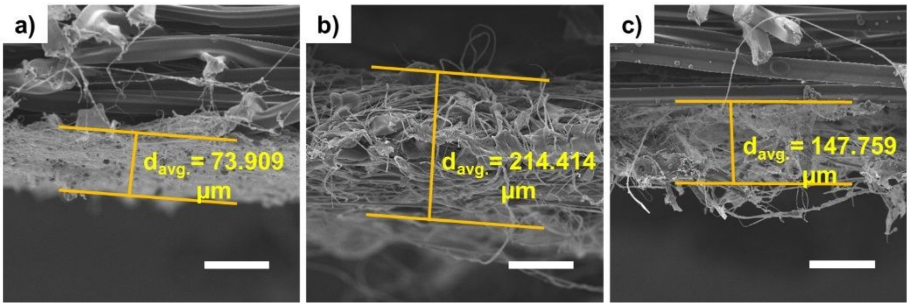

Figure S2. Comparative fiber diameter and pore size graphs of the SB-U-N, SB-U-M unimodal and SB-B bimodal webs.

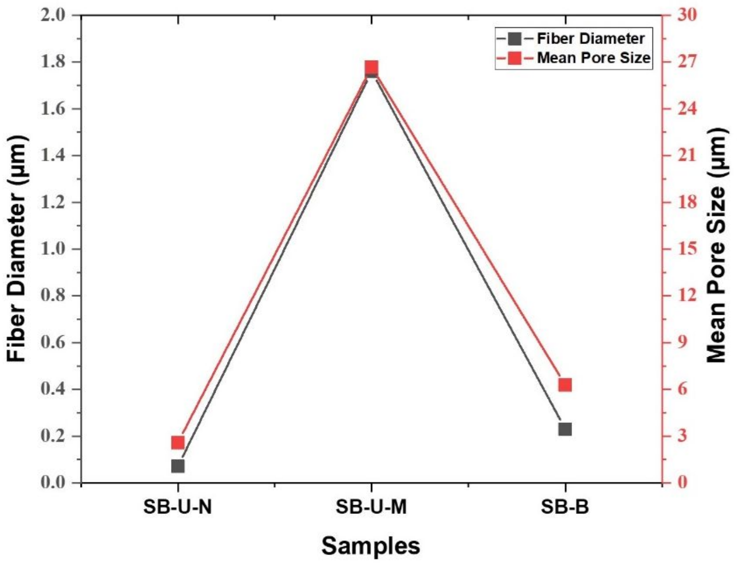

Figure S3. Flow/Pressure graphs of the a) SB-U-N, b) SB-U-M unimodal and c) SB-B bimodal webs.

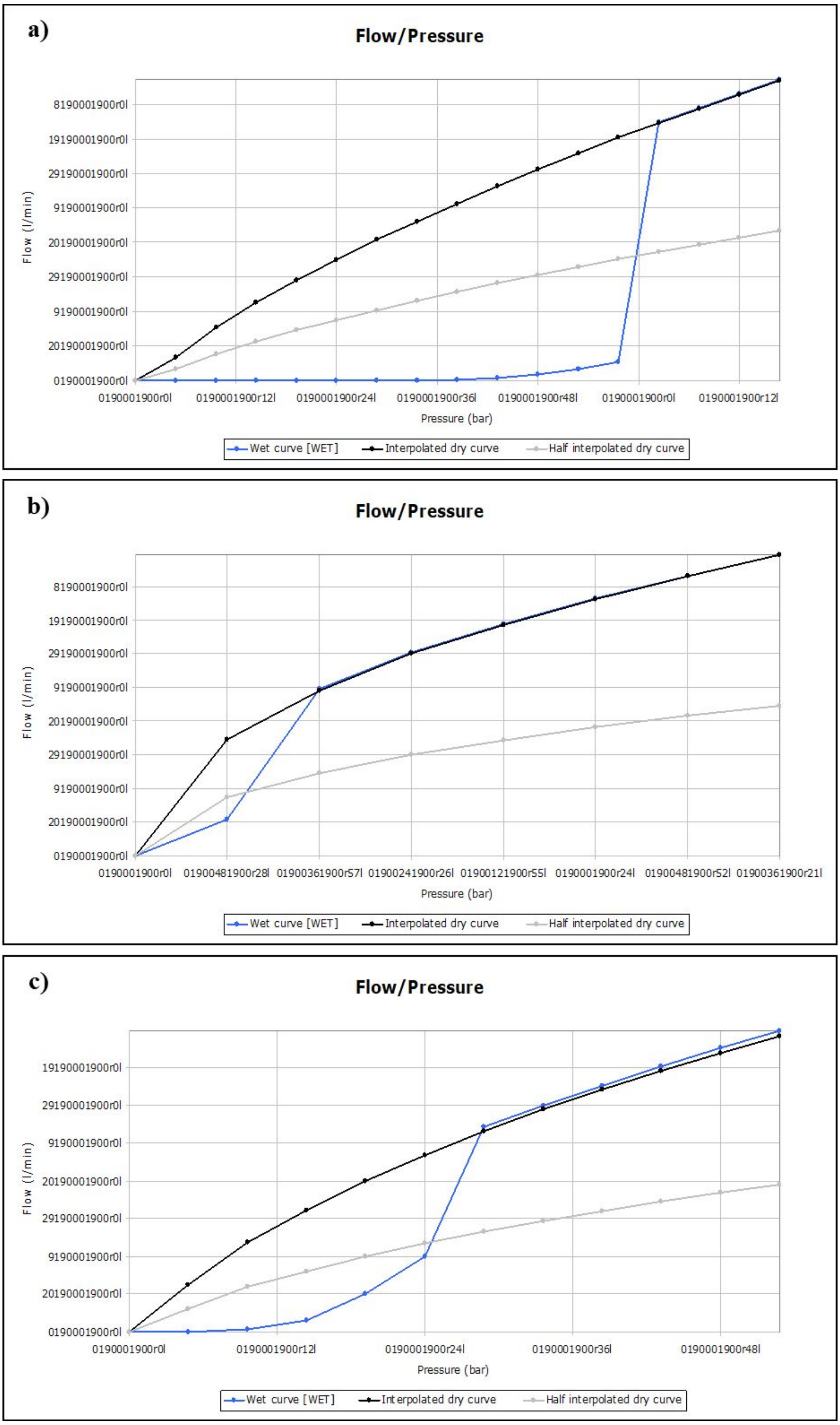

Table S1. Preliminary sample codes and variable system parameters during production.

Samples	Solution Concentration (%)	Feeding rate (ml h^{-1})	Air Pressure (bar)
SB 1.1	7	5	1
SB 1.2			2
SB 1.3		10	1
SB 1.4			2
SB 1.5		15	1
SB 1.6			2
SB 1.7	13	5	1
SB 1.8			2
SB 1.9		10	1
SB 1.10			2
SB 1.11		15	1
SB 1.12			2
SB 1.13	15	5	1
SB 1.14			2
SB 1.15		10	1
SB 1.16			2
SB 1.17		15	1

SB 1.18			2

Table S2. Viscosities of different concentration PA6 solutions

**Solution Concentration (wt.%)**	**Spindle Speed (rpm)**	**Spindle Diameter Code**	**Solution Viscosity (mPa.s)**
7	100	R2	71.6
13		R3	405.7
20		R4	1488.6

Table S3. The mechanical performance values of the webs

**Sample**	**Tensile Strength (MPa)**	**Breaking Elongation (mm mm^{-1})**	**Elastic Modulus (MPa)**
SB-U-N	0.0853	0.2097	0.004169
SB-U-M	0.1316	0.1447	0.016747
SB-B	0.1008	0.1981	0.012935
